# Parenting Under Pressure: Associations between Perceived Social Pressure and Parental Involvement among Mothers and Fathers

**DOI:** 10.1007/s10826-024-02945-5

**Published:** 2024-11-08

**Authors:** Gaëlle Venard, Grégoire Zimmermann, Jean-Philippe Antonietti, Cindy Eira Nunes, Stijn Van Petegem

**Affiliations:** 1 FAmily and DevelOpment research center (FADO), Université de Lausanne, Lausanne, Switzerland; 2 Centre de recherche de psychologie du développement, de la famille et des systèmes humains (DeFaSy), Université Libre de Bruxelles, Bruxelles, Belgium; 3 F.R.S.-FNRS Research Associate, Bruxelles, Belgium

**Keywords:** Adolescence, APIM, Gender, Parenting, Societal pressure

## Abstract

In many Western countries, the ideology of intensive parenting has gained prominence in the discourse of experts, policymakers, and within popular culture. This ideology emphasizes deep parental involvement in emotional, physical, and financial aspects (Lee et al., 2014). Meeting these demanding standards can exert significant pressure on parents, especially on mothers often considered as the primary caregiver. Moreover, these pressures may prompt parents to be highly, and potentially overly, involved in their children’s lives. Using data from 146 parent dyads (N = 292 parents; M_age_ = 47.57 years) of Swiss adolescents, the study explores parental perceptions of pressure to be a perfect parent and its association with one positive (responsiveness) and two negative types of involvement (overprotection and overvaluation). Thereby, we estimated Actor-Partner Interdependence Models (APIM) to examine mutual influences between mothers and fathers. The results indicated that mothers reported experiencing significantly more pressure than fathers. We found evidence for a positive association between perceptions of pressure and parental overprotection among both parents. The results also showed that there was a significant association between feelings of pressure and overvaluation, but only among fathers. Associations between pressure and responsiveness were not significant, and no significant partner effects were observed in any of the models. In conclusion, mothers particularly face heightened pressure to be perfect parents, but both parents may adapt their parenting strategies in response to perceived pressure to be perfect as a parent. These findings highlight the potential issues associated with societal pressures on parents and their impact on parenting behavior.

“I want the best for my child” is a sentence often said by parents. According to various media, parenting today can be compared to a performance (Faircloth, 2014). There are several indicators that may signal whether parents perform appropriately, such as their child’s success at school, their overall health, and the activities in which they are involved (Smyth & Craig, 2017). In fact, modern-day parents are expected to be mentally, financially, and physically highly involved to meet the demanding standards of what some sociologists call the culture of “intensive parenting” (Lee et al., 2014; Martin & Leloup, 2020). In the context of these changing societal expectations about parenthood, parents may more often feel pressured to adopt certain specific ways of parenting (e.g., Rizzo et al., 2013; Smyth & Craig, 2017). In addition, despite fathers’ greater involvement in their children’s upbringing (Schoppe-Sullivan & Fagan, 2020), mothers are more likely to feel pressure, as they are still seen as the primary caregiver (Hays, 1996; Meeussen et al., 2022; Faircloth, 2014), and therefore are more likely to be concerned about adopting a degree of parental involvement that meets the high societal standards (Shirani et al., 2012; Wall, 2010). Yet, the question of whether and how mothers’ and fathers’ perceptions of societal pressure are predictive of their parental involvement has rarely been empirically studied (Lee & Macvarish, 2020). In fact, despite the extensive body of research on parenting, there remains a notable gap in our understanding of how societal norms differentially influence maternal versus paternal involvement. Additionally, there is little empirical research among parents of adolescents that looks at the social pressure to be perfect as a parent, and its correlates in terms of parenting, with the exception of Wuyts and colleagues (2015), who highlighted a significant association between perceived social pressure and controlling parenting. Nevertheless, adolescence is a particular developmental period in terms of parenting, with specific developmental challenges for the child and for the parents accordingly. Notably, parents need to balance appropriate parental involvement and support with adolescents’ growing need for independence and autonomy (Smetana & Rote, 2019; Smetana et al., 2015). That is, even though parental involvement remains essential, certain types of involvement (e.g., overprotection) may not always be appropriate and could potentially lead to mental health difficulties for both adolescents (e.g., LeMoyne & Buchanan, 2011; Van Petegem et al., 2020) and parents (e.g., Zimmermann et al., 2022).

The goal of this study is therefore to examine associations between perceived pressure to be a perfect parent among dyads of parents of adolescents, and three dimensions of parenting that reflect parental involvement. Specifically, we examined relations with parental responsiveness, which is considered a type of involvement that is attuned to the child’s developmental needs (Bogenschneider & Pallock, 2008), alongside with parental overprotection and overvaluation, which are considered as less attuned due to their excessive nature (Brummelman et al., 2015; Rousseau & Scharf, 2015). We further used Actor-Partner Interdependence Models (APIM) to examine mutual influences between mothers and fathers. By exploring these research questions, we aimed to shed light on the ways in which societal norms may affect parenting in adolescence, while also examining potential differences between mothers and fathers. Additionally, by identifying factors that may drive types of parental involvement, we hope to gain a deeper insight into the dynamics of parenting and to offer opportunities for mitigating inadequate parenting behaviors.

## The Pressure to be a Perfect Parent

Since the 1940s and 1950s, scientific research has begun to take a great interest in child welfare and parenting (e.g., Spock, 1946). In this context, the literature on parenting has grown considerably, which has contributed to a redefinition of the concept of parenting (Lee et al., 2014). That is, parenting no longer corresponds to the natural activity of caring for the child, but now refers to all activities prescribed for children’s well-being and their optimal development (Lee et al., 2014; Martin & Leloup, 2020). In sociological accounts, it is argued that this redefinition of childrearing may have contributed to the emergence of the ideology of intensive parenting, which was originally termed “intensive mothering” due to its specific targeting of mothers (Hays, 1996; Lee et al., 2014; Wall, 2010). This ideology promotes the idea that to be considered ‘fit’ for parenting, parents should be well-informed about all expert advice and are encouraged to invest a great deal of their time, energy, and financial resources (Arendell, 2000). On top of that, their parenting would play an essential role in the healthy development of their child — hence, according to this ideology, parents would also become the primary responsible figures for the development of the society of tomorrow more broadly (Bernstein & Triger, 2010; Hays, 1996; Lee et al., 2014; Martin & Leloup, 2020). This responsibility for their child’s education and success is posited to rest entirely on the parents’ shoulders, suggesting that outcomes are almost exclusively determined by their actions, and is therefore sometimes referred to as “parental determinism” (Lee et al., 2014; Lee & Macvarish, 2020; Martin & Leloup, 2020). In this context, parents may feel an overwhelming pressure to achieve perfection in their parenting roles, a burden that may be especially pronounced for mothers, who are still considered as the primary caregiver (Hays, 1996; Meeussen & Van Laar, 2018).

## Parents under Pressure: Gender Disparities

Historically, the field of developmental psychology primarily has focused on mothers, often putting aside the role of fathers (Coltrane, 2000). Early theories (e.g., psychoanalysis, attachment theory) generally portrayed mothers as mainly responsible for both the physical and psychological health of the child. This emphasis on mothers may have fueled the ideology of intensive mothering, which posits that women are naturally better equipped for childcare and, therefore, make better parents than their male counterparts (Coleman et al., 2007; Hays, 1996). Consequently, despite fathers’ greater involvement and engagement in their children’s lives today compared to a few decades ago (Schoppe-Sullivan & Fagan, 2020; Shirani et al., 2012; Sullivan, 2010), paternal figures are often considered as less affected by the ideology of intensive parenting (Faircloth, 2020; Shirani et al., 2012).

On their side, in the context of an intensive mothering ideology, women are expected to exhibit their dedication as a mother by being highly involved in their children’s lives or even resigning from their employment (Faircloth, 2020; Ranson, 2004; Wolf, 2016). In fact, no mental or physical sacrifice appears too great for mothers in their pursuit of optimizing the child’s development (Hays, 1996; Wolf, 2016). Given these expectations, mothers often experience more pressure than fathers regarding how they should fulfill their parental role (Meeussen & Van Laar, 2018). Several studies indicated that guilt is a common feeling among women, especially when they aspire to perfectly adhere to the norms of intensive mothering (Meeussen & Van Laar, 2018; Sutherland, 2010; Wall, 2010). To counteract these guilty feelings, mothers of young children who endorse these intensive mothering standards have been found to adopt highly involved parenting practices, that is, they tend to commit vast amounts of time and engage intensively with their children, often at the expense of their own needs and desires (Wall, 2010). In addition, even as children reach adolescence, mothers tend to dedicate more time than fathers to emotional support and behavioral control (Mastrotheodoros et al., 2019), suggesting that gender disparities persist during this developmental phase. However, there is a scarcity of research examining the association between parents’ perceptions of pressure to be a perfect parent and their involvement during this developmental period, which is unfortunate, as parents need to readjust their level of involvement to adolescents’ changing developmental needs during this transitional phase in life (Gutman & Eccles, 2007).

## Parental Involvement during Adolescence: Between Responsiveness, Overprotection, and Overvaluation

As illustrated earlier, past research indicates that ambient societal norms and pressures about parenthood may become apparent through parents’ specific parent practices (e.g., Wall, 2010; Wuyts et al., 2015). In this study, in order to highlight potential manifestations of parental involvement, we focused on three different types of parenting: responsiveness, overprotection, and overvaluation. These three types of involvement differ in terms of quality; whereas the first dimension (i.e., responsiveness) is considered to be attuned and beneficial for adolescents’ development (e.g., Davidov & Grusec, 2006), the latter two dimensions (i.e., overprotection and overvaluation) are considered unattuned and potentially harmful for adolescents’ development (e.g., Brummelman et al., 2015; Van Petegem et al., 2020).

Responsiveness is an adaptive parenting dimension and is defined as the extent to which parents are attuned, supportive, and acquiescent to children’s needs and demands (Baumrind, 1991). That is, when a child is upset or distressed, responsive parents have sensitive reactions such as comforting or helping (Gottman et al., 1996). When parents are generally responsive, children are more likely to develop a sense of security and self-confidence, social competence, and adaptive coping skills (Davidov & Grusec, 2006; Kiss et al., 2014). Among adolescents, parents’ responsiveness was found to be positively associated with adolescents’ responsible behavior (i.e., completing chores, following through on promises, showing good judgment when spending money or choosing friend; Bogenschneider & Pallock, 2008) and less externalizing problems (Pinquart, 2017).

However, despite good intentions, parents can also provide attention and protection that is excessive, considering the child’s developmental level (Thomasgard et al., 1995; Venard et al., 2023). This parenting dimension, referred to as parental overprotection, can manifest through a variety of parenting practices, such as when parents constantly warn about potential dangers and are excessively preoccupied about the adolescent’s safety (Brenning et al., 2017; Omer et al., 2016), when they solve problems prematurely by providing help when this is not requested (Segrin et al. 2013), or when they intrude upon the adolescent’s privacy (Hawk et al., 2009). Higher levels of parental overprotection during adolescence and young adulthood were generally found to relate to lower psychosocial adjustment, including higher levels of distress, lowered self-esteem, excessive worries about relationships, and unassertive interpersonal behavior (LeMoyne & Buchanan, 2011; Mathijs et al. 2024; Rousseau & Scharf, 2015).

Parents who adhere to an intensive parenting ideology may also hold the belief that their child merits special treatment and attention (Brummelman et al., 2017; Twenge & Campbell, 2009). Parental overvaluation refers to a parent’s belief that their child is superior to others, a tendency to overestimate their child’s capacities, and therefore is entitled to privileges (Brummelman et al., 2015; Brummelman & Sedikides, 2020). Such unrealistically positive views of their children may also be expressed through inflated praise, where parents praise their children in excessive ways to bolster their child’s self-esteem (Thomaes and Brummelman (2016); Twenge, 2006). However, both overvaluation and inflated praise may be detrimental for the children, putting them at risk for developing a fragile self-esteem and narcistic traits. Indeed, overvaluation can be detrimental, as children’s narcissism can take root in such a family climate (Brummelman et al., 2015). Feeling more gifted than others, narcissistic children would crave other people’s admiration to feel good about themselves (Thomaes et al., 2008). Hence, children who are overvalued by their parents might become dependent on external validation to feel worthy, and are therefore at risk for developing psychopathology (Thomaes et al., 2009, 2013).

In spite of little research among parents indicating that ambient societal norms and pressures about parenthood may become apparent through parents’ specific parent practices (e.g., Wall, 2010;Wuyts et al., 2015), we are not aware of existing research examining associations between perceived societal pressure and parental responsiveness, overprotection and overvaluation among parents of adolescents It appears significant to understand whether the pressure to be a perfect parent affects the quality of involvement, especially during adolescence due to the potential harmful consequences (eg. Brummelman et al., 2015; Van Petegem et al., 2020). In addition, to fully comprehend intensive parenting and its correlates with parental involvement, it also seems important to explore the dyadic dynamics between mothers and fathers.

## Mutual Influences between Mothers and Fathers

The family is a system of mutual influences (Cox & Paley, 2003; McHale & Lindahl, 2011). Both parents may have raised jointly their children and they may often interact about upbringing, co-constructing their beliefs and practices (McHale & Lindahl, 2011). As a consequence, the characteristics (e.g., beliefs, experiences, parenting behaviors) of one parent may influence the characteristics of the other parent (McBride et al., 2005; Ponnet et al., 2013; Rousseau & Scharf, 2015). In this respect, past research has highlighted such interdependence between parents and the possibility of carry-over effects between family members (e.g., Brenning et al, 2017; Peterson et al., 2008). For instance, Brenning et al. (2017) found that maternal anxious attachment representations are predictive of their partner’s separation anxiety and overprotective parenting, suggesting interdependence between the two parental figures. Furthermore, Ponnet et al. (2013) showed that higher levels of maternal stress were negatively linked to their partner’s responsiveness toward their children. Thus, it may be expected that feelings of pressure from one parent may be predictive of the other parent’s involvement. However, we are not aware of any previous studies examining how one parent’s perceptions of social pressure are related to their other parent’s parenting, in terms of responsiveness, overprotection, and overvaluation. Therefore, we aim to overcome the conceptualization of parental beliefs and experiences, such as parents’ perceptions of pressure to be a perfect parent, as uniquely intra-individual, by also considering dynamics that may play between parental figures.

## The Present Study

This study has been conducted in a sample of Swiss parents of adolescents. In Switzerland, gender disparities are still prevalent in the private sphere. For instance, mothers are more likely than fathers to be in charge of the household (60% of mothers vs. 6% of fathers) and the care for children (48% of mothers vs. 6% of fathers; OFS, 2019, 2021). As a consequence, it seems important to examine potential differences between mothers and fathers when considering the role of pressure to be a perfect parent in the prediction of parental involvement during adolescence.

First, we wanted to find out whether mothers of adolescents were more sensitive to the pressure to be a perfect parent, and whether their level of involvement differed significantly from fathers. To do so, we examined mean-level differences between mothers and fathers in terms of perceived pressure to be a perfect parent, as previous studies investigating parents’ pressure to be perfect most often only focused on mothers (Meeussen et al., 2016; Meeussen & Van Laar, 2018; Sutherland, 2010). As Western societies continue to consider mothers as the primary caregiver (e.g., Hays, 1996; Wall, 2010), we expected that mothers would perceive more pressure than fathers. The mean-level differences between mothers and fathers for responsiveness, overprotection, and overvaluation, were investigated as well. Considering previous studies (Brummelmann et al., 2015; Davidov & Grusec, 2006; Rousseau & Scharf, 2015; Schiffrin et al., 2019), we hypothesized that there would be no differences between mothers and fathers concerning responsiveness and overvaluation, but that overprotection would be reported more by mothers than by fathers. Second, we used APIM to estimate associations between perceived pressure and parental involvement, thereby considering both *actor* effects (i.e., associations between a parent’s pressure perceptions and their own parenting) and *partner* effects (i.e., associations between a parent’s pressure perceptions and their partner’s parenting). For actor effects, to our knowledge, no research has examined associations between pressure to be a perfect parent and the parental dimensions of responsiveness, overprotection, and overvaluation from a gender perspective. However, we expected that parents’ pressure perceptions would predict higher scores on all three dimensions, and this association would be particularly pronounced among mothers in comparison to fathers (Meeussen & Van Laar, 2018). For partner effects, drawing upon family systems theories (McBride et al., 2005), we predicted reciprocal relations between mothers and fathers where one parent’s perceived pressure is predictive of their partner’s parental involvement.

## Method

### Sample and Procedure

Participants were parents of adolescents in their last year of obligatory schooling in the French-speaking part of Switzerland. After obtaining approval from the School and Youth department of the Canton of Vaud, we contacted sixteen schools in the area of Lausanne and nine accepted to be part of the study. After obtaining a pre-arranged date by phone or e-mail, schools were visited by two trained members of the research team. They first explained the overall purpose of the study during a class period, as well as the voluntary nature of participation and the confidential treatment of the data. Then, they distributed envelopes among pupils, which contained two informed consents, two questionnaires and two pre-stamped envelopes. The pupils were instructed to give the questionnaires to their parents (or the two persons they considered as most involved in their education), regardless of whether they were in a relationship or not. The general goal of the study, the voluntary nature of participation and the confidential treatment of the data were described in the informed consent. Parents were invited to fill out the questionnaires separately, and to send the questionnaire back within three weeks in separate envelopes. The questionnaires had a unique and randomly generated code, which allowed us to link the data of the parents of the same adolescent. All participating parents were rewarded with 20 Swiss francs (CHF; approximately equivalent to 23 US$; 1CHF = 1.14 US$) voucher in a local store. The study followed the ethical standards of the Swiss Society of Psychology (SSP) and was approved by the Coordinating Committee for educational research of Vaud.

Our total sample consisted of 467 parents, composed of 283 mothers (60.6%) and 184 fathers (39.4%), with a mean age of 47.28 years (*SD* = 5.46). However, as the analyses focused on dyadic dynamics between father-reported and mother-reported variables, only complete dyads were included in our analyses, resulting in a sample of 146 dyads of parents of adolescents (*N* = 292 parents; 62.5% of the initial sample). The mean age of mothers and fathers was 45.80 years (*SD* = 4.51, median = 46, min_age_ = 32, and max_age_ = 56) and 49.30 years (*SD* = 6.10, median = 49, min_age_ = 34, and max_age_ = 70), respectively. With regard to nationality, most parents were Swiss citizens (71.9% and 69.9% of mothers and fathers respectively) or from European Union countries. The majority of parents reported being married or living together (79.5%), whereas 20.5% reported being divorced or separated. In the sample, the annual households’ income and the parents’ educational level of the sample appears to mirror that of the broader population residing in the Canton. In this area of Switzerland, the median gross household income is 139'541 CHF for households with two children (Statistique Vaud StatVD, 2015) We consider couples earning less than 88’000 CHF with two children to be low-income couples and those earning more than 190’000 CHF to be high-income couples. There was a large variety in the annual household income, with 31.3% earning less than 88'000 CHF, 13.4% between 88’000 and 103'000 CHF, 15.7% between 103'000 and 146'000 CHF, 14.2% between 146'000 and 190'000 CHF, and 9.7% above 190’000 whereas 15.7% either did not report or indicated preferring not to report their household income. Concerning parents’ educational level in this area, more than 40% percent of the adults have on average a university degree or a bachelor in specialized training (other forms of education), which corresponds to our sample. Specifically, the distribution was as follows: 15.2% had completed only mandatory schooling, 31% had received professional or vocational training, 8.8% had obtained a baccalaureate, 34.5% had a university degree, and 10.3% had pursued other forms of education. Concerning employment, most of the parents reported being employed or independent (83%). While 91,4% of fathers worked full time (80% - 100%), the majority of mothers worked part-time (63.0%). In our sample, 12.0% of the parents reported having one child, 55.8% had two children, and 32.2% reported having three or more children. Parents filled out questionnaires with respect to their adolescent child in the last year of obligatory schooling. These adolescents were, on average, 14.7 years of age (*SD* = 0.61), 60.3% were girls, and most adolescents followed either vocational education (23.1%) or high school education (75.5%).

## Measures

Participants filled out French versions of questionnaires, which were either available or translated following the recommendations of the International Test Commission (Hambleton, 2013). Items were scored on five-point Likert scales, ranging from 1 (*strongly disagree*) to 5 (*strongly agree*).

### Pressure to be a Perfect Parent

Parents’ perception of pressure to be a perfect parent was measured through two items from the study of Meeussen and Van Laar (2018), which were inspired by the literature on idealized motherhood (Henderson et al., 2016). Participants reported their feelings of pressure to be a perfect parent by rating to what extent they agreed with the statements: “I feel pressured to be ‘perfect’ in my role as a parent” and “My social environment sets very high expectations for me as a parent to live up to”. In this study, Cronbach’s α’s was 0.76 for mothers and 0.70 for fathers.

### Parental Responsiveness

Parental responsiveness was measured through the Acceptance-Rejection subscale of the Child Report of Parenting Behavior Inventory (CRPBI; Davidov & Grusec, 2006; Schaefer, 1965). This subscale includes 7 items that measure the degree to which parents show warmth and affection and are sensitive when the child is distressed. Initially, the scale was designed to be completed by children, but as in previous research (e.g., Brenning et al., 2017), the items were slightly revised to make them amenable to parent self-report (e.g., “My child feels better after discussing his worries with me” or “I smile at my child very often”). In this study, Cronbach’s α’s was 0.76 for mothers and 0.80 for fathers.

### Parental Overprotection

Parental overprotection was assessed using the 10-item Anxious Overprotection subscale of the short version of the Multidimensional Overprotective Parenting Scale (S-MOPS; Chevrier et al., 2023; Kins & Soenens, 2013). This scale assesses several aspects of overprotective parenting, including parents’ anxious rearing, premature problem solving, infantilization, and privacy invasion. Example items include “I am all over my child” and “I follow everything my child does, even when he/she needs time to himself/herself”. The psychometric properties of this short version are provided by Chevrier et al. (2023). Good internal consistency was observed in this study, with Cronbach’s alphas of 0.83 and 0.80 respectively for mothers and fathers.

### Parental Overvaluation

Parents rated the Parental Overvaluation Scale (POS; Brummelman et al., 2015). This scale assesses the tendency of parents to overvalue their child. The POS is a 7-item instrument that examines the degree to which parents think their child is extraordinary (e.g., “My child deserves special treatment” or “I would not be surprised to learn that my child has extraordinary talents and abilities”). The psychometric properties of the POS are provided by Brummelmann et al. (2015), who found a strong stability and validity of the items throughout several studies. In the present study, Cronbach’s α were 0.77 for mothers and 0.75 for fathers.

### Data Analysis

All statistical analyses were performed in the R environment version 4.1.2 (R Development Core Team, 2023). Raw data were structured as dyadic data. In other words, the data were organized in a pairwise structure so that each line represented a dyad containing the mother’s and father’s scores. Concerning our variables of interest, only 1.1% of data were missing. For handling missing data, we used Hot deck imputation, which is a method replacing each missing value with an observed response from a “similar” responding unit; the data set is sorted and missing values are imputed sequentially running through the data set line (observation) by line (observation) (Kowarik & Templ, 2016).

The preliminary analyses involved examining descriptive statistics (means and standard deviations) and correlations between the variables of interest. To examine the potential impact of socio-economic status on our results, we also verified the correlations between our target variables and the parents’ educational level and annual household income. We subsequently controlled for significant effects in the main analyses.

As for our main analyses, we first tested mean level differences between mothers and fathers using Repeated Measures MANOVA, with parental gender as a between-subject independent variable, and parental responsiveness, overprotection, and overvaluation as dependent variables. We also calculated Cohen’s *d* to assess the effect size. Cohen (1988) suggested that *d* = 0.20 be considered a “small” effect size, 0.50 represents a “medium” effect size and 0.80 a “large” effect size. Then, we conducted several APIMs, using a structural equation modeling (SEM) framework, to test three models, one for each parental dimension. For each model, the approach was the same: first, we examined the saturated model with the degree of freedom and the chi-square value at 0. Second, we constrained the actor and partner links that were not significantly different from 0. Third, to test whether the actor or partner effects differed significantly between mothers and fathers, we specified equality constraints across mothers and fathers. When there is a statistically significant change in the chi-square value as compared with the model with no equality constraints, this indicates that actor or partner effects were statistically different from each other. A nonsignificant change in the chi-square value as compared with the model with no equality constraints indicates no differences in the strength of the associations for mothers vs. fathers. Model fit was assessed by the chi-square test, the chi-square to *df* ratio (*χ*^2^/*df*), the root mean square estimation of association (RMSEA), the standardized root mean squared residual (SRMR), and the Bentler comparative fit index (CFI: Barrett, 2007; Cheung & Rensvold, 2002). Good model fit was indicated when *χ*^2^/df is lower than 3.0, an RSMEA lower 0.06 ( > 0.10 suggests poor fit), SRMR under 0.08 and CFI larger than 0.95 (Hu & Bentler, 1999).

## Results

### Preliminary Analyses

Table 1 presents descriptive statistics of, and correlations between, the study variables. First, mothers’ and fathers’ pressure to be a perfect parent correlated significantly positively, as was the case for mothers’ and fathers’ overprotection and overvaluation. Further, as presented in Table 1, mothers’ pressure to be a perfect parent correlated positively with maternal overprotection (moderate effect size), whereas fathers’ pressure to be a perfect parent correlated positively with paternal overvaluation (moderate effect size). Regarding socio-economic status, there was a significant negative correlation between both mothers’ and fathers’ education level and paternal overprotection. No significant correlation was observed between the parents’ annual income and the variables of interest.

**Table 1:** Means, Standard Deviations and Correlations among the Study Variables

	*M*	*SD*	1.	2.	3.	4.	5.	6.	7.	8.	9.	10.	11.
1. Perceived pressure (M)	2.93	1.11											
2. Perceived pressure (F)	2.48	0.95	0.25^**^										
3. Parental responsiveness (M)	4.37	0.45	−0.09	0.06									
4. Parental responsiveness (F)	4.26	0.51	0.00	−0.03	−0.00								
5. Parental overprotection (M)	1.90	0.56	0.20^*^	0.09	−0.01	0.00							
6. Parental overprotection (F)	1.77	0.48	−0.00	0.16	−0.03	−0.14	0.33^**^						
7. Parental overvaluation (M)	2.31	0.73	0.12	0.11	−0.07	0.10	0.35^**^	0.22^**^					
8. Parental overvaluation (F)	2.38	0.76	0.15	0.22^**^	0.00	−0.10	0.24^**^	0.25^**^	0.42^**^				
9. Education level (M)	3.19	1.55	0.13	0.02	−0.06	0.08	−0.11	−0.28**	0.04	−0.04			
10. Education level (F)	3.29	1.50	0.12	0.05	−0.15	−0.00	−0.15	−0.27**	−0.04	−0.08	0.58**		
11. Annual income (M)	6.29	2.91	0.08	0.02	−0.00	−0.03	0.04	−0.07	−0.03	−0.07	0.29**	0.24**	
12. Annual income (F)	6.88	2.54	−0.01	−0.04	−0.10	−0.03	−0.12	−0.03	−0.13	0.03	0.19*	0.46**	0.38**

M = mother, F = father

^*^p < 0.05. ^**^p < 0.01

### Main Analyses

Then, we examined differences between mothers and fathers on the variables of interest. The Repeated Measures MANOVA, which examined mean-level differences between mothers and fathers, yielded a significant multivariate effect, *F*(1,145) = 16.86, *p* < 0.001. Subsequent univariate analyses indicated that mothers, compared to fathers, reported higher levels of pressure to be a perfect parent, *F*(1,145) = 18.38, *p* < 0.001, *d* = 0.36. The difference for parental responsiveness was not statistically significant, *F*(1,145) = 3.73, *p* = 0.055, *d* = 0.16, but was statistically significantly higher among mothers for parental overprotection, *F*(1,145) = 5.82, *p* < 0.05, *d* = 0.20. There was no significant differences between mothers and fathers for overvaluation, *F*(1,145) = 1.29, *p* = 0.258, *d* = 0.09.

**Fig. 1:**
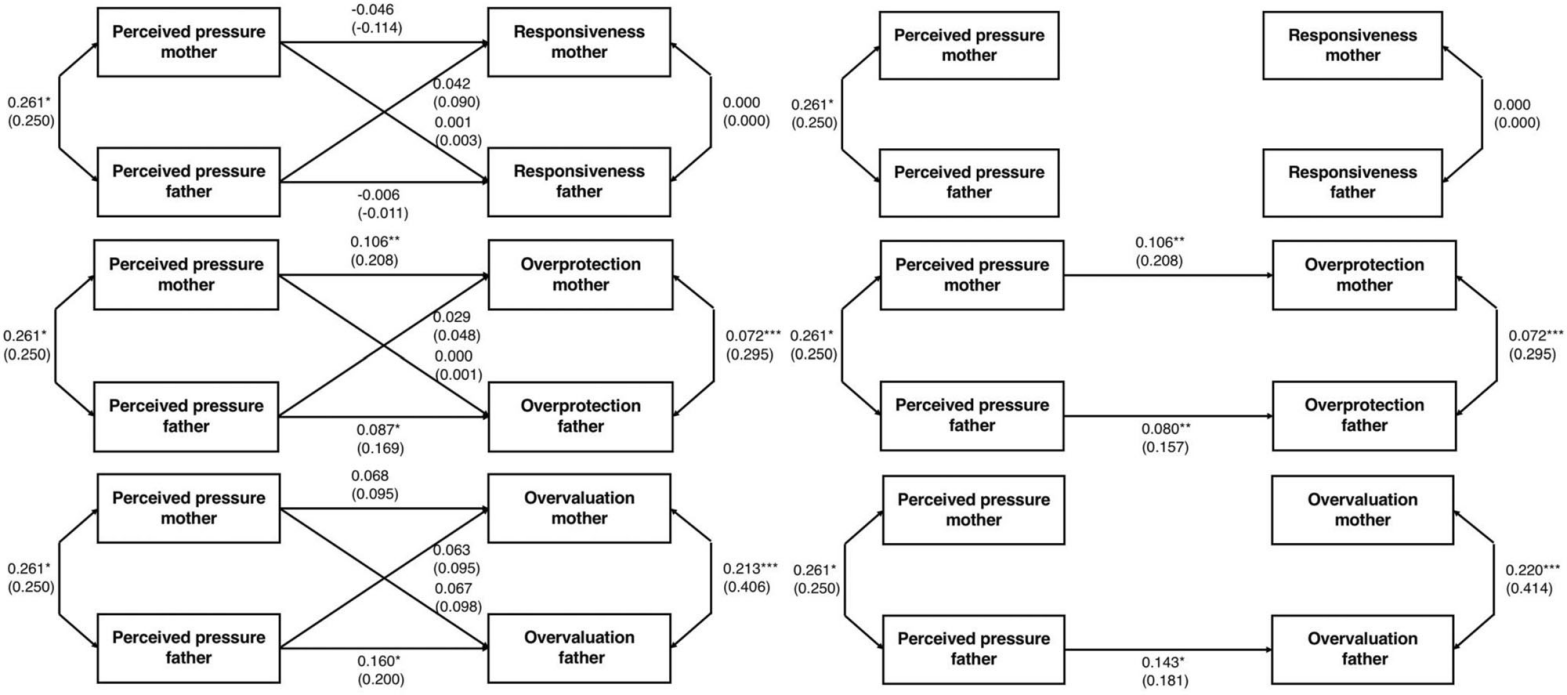
Actor-Partner Interdependence Models without constraints (left column) and with constraints (right column). Standardized coefficients are presented between brackets. *p < 0.05, **p < 0.01, ***p < 0.001

Figure 1 depicts the results of the APIM, showing the saturated models and the most parsimonious models. The most parsimonious model for responsiveness had all actor and partner effects set to 0. This restricted model had a good fit, *χ*^2^(4) = 2.49, *p* = 0.65, *χ*^2^/df = 0.62, CFI = 1.00, RMSEA = 0.000 [90%CI:0.000-0.103], SRMR = 0.035, and it did not differ significantly from the initial model (Δ*χ*^2^(4) = 2.49, *p* = 0.65). The models revealed that there were no actor effects and no partner effects between pressure to be perfect and parental responsiveness. For overprotection, we controlled for the educational level of both parents, as it was significantly correlated with paternal overprotection. The partner effects were nonsignificant, and were therefore constrained to 0. Then, the actor effects were constrained to be equal across mothers and fathers. The final model had a good fit: *χ*^2^(6) = 3.84, *p* = 0.70, *χ*^2^/df = 0.64, CFI = 1.000; RMSEA = 0.000 [90%CI:0.000-0.081], SRMR = 0.045. This final model did not differ significantly from the initial model (Δ*χ*^2^(2) = 0.35, *p* = 0.84). As presented in Fig. 1, we found a positive association between perceived pressure and parental overprotection for both mothers and fathers. Thus, although mothers report significantly more pressure to be a perfect parent (i.e., mean-level differences in perceived societal pressure), this pressure is similarly associated with overprotection for both mothers and fathers. Finally, for overvaluation, in the fully saturated model, only the father’s actor effect was significant. The two partner effects and the mothers’ actor effect therefore were constrained to 0. The constrained model showed excellent fit to the observed data: *χ*^2^(3) = 3.68, *p* = 0.30, *χ*^2^/df = 1.23, CFI = 0.98; RMSEA = 0.040 [90%CI:0.000-0.152], SRMR = 0.063. This final model did not differ significantly from the initial model (Δ*χ*^2^(3) = 3.68, *p* = 0.30). As can be noted in Figure I, we observed a significant positive association between feelings of pressure and overvaluation, but only among fathers.

## Discussion

In our contemporary society, the benchmarks for parenting have escalated, requiring a greater degree of parental involvement, for those who wish to adhere the principles of intensive parenting ideology. In this study, we used APIM to examine within a dyadic framework the links between perceived social pressure to be a perfect parent among mothers and fathers and three dimensions that reflect parental involvement (i.e., responsiveness, overprotection, and overvaluation). In addition to examining gendered differences, this research investigated how mothers’ and fathers’ feelings of pressure to be a perfect parent were associated with their own and their partner’s parental responsiveness, overprotection, and overvaluation during adolescence.

First, descriptive analyses showed that mothers perceived significantly higher levels of pressure to be a perfect parent compared to fathers. Mothers also reported significantly higher levels of overprotection, although the difference was small, considering Cohen’s *d*. No statistically significant differences were found for overvaluation and responsiveness. These mean-level gender differences regarding perceived societal pressure are in line with our hypothesis that mothers are more susceptible to experience such pressure. Thus, mothers are still seen as the primary caregiver, in spite of societal trends where the division of household tasks is becoming relatively more egalitarian (Mannino & Deutsch, 2007; Monna & Gauthier, 2008; Smyth & Craig, 2017). This seems to suggest that women generally internalize socially constructed ideals of motherhood more often as a key aspect of their identity and are socially expected to be the “experts” in care, while fathers who are sensitive to intensive parenting would be inclined to focus more on their role as financial providers (Meeussen et al., 2022; Shirani et al., 2012).

This study seems to reveal that constructed ideals of motherhood persist beyond early childhood, even when the developmental needs evolve as the child becomes an adolescent. Notably, the period of adolescence is characterized by an increasing need for independence and autonomy, more time spent outside parental home, more experimentation and exploration of different identity alternatives (Smetana & Rote, 2019; Smetana et al., 2015; Zimmermann et al., 2017). During this developmental period, it is important for parents to provide structure through rules and to offer support when their adolescents are confronted with problems or distress, but they but they must not warn them of the slightest danger, nor provide help without their asking for it (Smetana & Rote, 2019). Thus, even though adolescents no longer need the same type of involvement and presence, maternal figures still feel the pressure to live up to the standards of a perfect mother. This result is important to consider because ideals of motherhood can be distressing for women, leading them to feel guilty (Maclean et al., 2021) and distressed (Henderson et al., 2016) and may potentially lead to burnout symptoms (Lin et al., 2021; Meeussen & Van Laar, 2018), especially if they perceive adolescence as a difficult period (Zimmermann et al., 2022).

Concerning the role of socio-economic status, descriptive analyses revealed that there was no significant correlation between annual household income and our variables of interest. However, we observed a notable negative association between paternal overprotection and the educational level of both parents. This finding aligns with previous studies suggesting that parents from lower socio-economic status are particularly sensitive to the norms of intensive parenting (Ishizuka, 2019), potentially leading to overprotective parenting (Toscano et al., 2022). These findings also may echo those of Wuyts et al. (2015), who indicated that parents dissatisfied with their own achievements might compensate by excessively investing in their children’s education. Potentially, these results may indicate that fathers may become overprotective as a means of ensuring his child to achieve the goals that he himself (or his partner) was unable to reach, as a means of projecting their unfulfilled dreams onto their children (cf. Wuyts et al., 2015b). However, future research is needed to examine this hypothesis explicitly.

Further, APIM analyses allowed us to determine the links between perceived pressure to be a perfect parent and three types of parental involvement, but also the association between the pressure to be a perfect parent of one parent and parental involvement of the other parent. A number of notable findings emerged. First, the results indicated that there was a positive association between feelings of pressure and parental overprotection for both parents, and there was no significant gender difference concerning this actor-oriented effect (i.e., gender did not moderate the association). This suggests that parents who experience pressure to be perfect as a parent, tend to overprotect their adolescent child to meet the norms of intensive parenting, irrespective of whether they are a mother or father. These parents probably find it more difficult to strike the right balance between being intensively involved with their adolescent child to support their development and, at the same time, giving them space to develop independently (Lee et al., 2014). During adolescence, this balance may be particularly difficult to find, as the child needs autonomy while at the same time needing parental figures to rely on (Smetana & Rote, 2019). More generally, this finding highlights the importance of considering the macro-context to better understand the phenomenon of parental overinvolvement, as it was previously found to relate to the way in which parents experience societal expectations and norms about how one ought to raise a child (Grolnick, 2003). Consistent with our results, Wuyts and colleagues (2015) suggested that when parents perceive external expectations regarding their children’s achievements, they are more likely to exert additional pressure on their offspring to excel. Thus, in response to norms of intensive parenting, parents may be also particularly inclined to overdo by engaging in overprotective parenting, which could generate anxiety and depressive symptoms in adolescents (Venard et al., 2023; Van Petegem et al., 2020, 2022).

Further, the APIM exploring the relationship between pressure and overvaluation also provided evidence for actor-oriented patterns, but only among fathers. As they experience pressure to be a perfect parent, fathers thus seem to hold the belief that their child is outstanding and deserves special attention (Brummelman et al., 2017; Twenge & Campbell, 2009). In line with these results, a study of middle- to upper-class fathers in India particularly highlighted how fathers directly associate their involvement as fathers with their child’s success (Sriram & Sandhu, 2013). Thus, for fathers, the impression of being a perfect parent would be reflected in the child’s performance and social and cognitive abilities (Smyth & Craig, 2017; Sriram & Sandhu, 2013). Fathers’ particular emphasis on child success may stem from the still-prevalent male gender stereotype associated with agency (e.g., ambitious, assertive, competitive) characterized by self-mastery and goal achievement (Eagly et al., 2020). For future research, it would be interesting to also explore qualitatively how motherhood and fatherhood are perceived by parents of teenagers in a European context.

By contrast, we found no evidence for an association between pressure to be a perfect parent and responsiveness. These results suggest that responsiveness, which is a positive aspect of parenting, is not associated with the way in which parents perceive societal expectations about parenthood and the pressure that may result from such expectations. In fact, as is shown in previous studies and our study, stress and pressure would rather induce inadequate involvement from parents (e.g., Yatziv et al., 2018). Potentially, the behaviors and beliefs of parents who perceived high pressure to be a perfect parent may be particularly driven by their concern for competence and excellence, instead of being guided by what the child would really need in terms of developmental and psychological needs (Dieleman et al. 2020). Their concern about excellence would be an obstacle to reflect on the adolescent mental world and they would find it difficult to behave accordingly (Dieleman et al., 2020).

Finally, contrary to our expectations, we observed no partner effects in any of the models. Thus, mothers’ and fathers’ feelings of pressure to be a perfect parent were unrelated to their partner’s parental responsiveness, overprotection, and overvaluation. These findings are in contradiction with a systemic conception of the family, which would suppose that parental figures influence one another and are affected by their partners in their parenting attitudes (McHale & Lindahl, 2011; Guay, et al., 2018). However, partner effects might still exist, but they might be too inconsistent across families to produce interpretable main effects. For example, while some parents may respond to their partner’s pressure to be a perfect parent by also becoming more overprotective, others may tend to engage in less overprotective behaviors, possibly to compensate for their partner’s overprotection (Zimmermann, et al., 2022). An alternative explanation for the absence of partner effects could be that feelings of pressure are particularly experienced individually, and do not necessarily reflect expectations regarding the partner. In fact, the items used to evaluate the pressure to be a perfect parent refer directly to the individual feelings of the parent (i.e., “I feel pressured to be ‘perfect’ in my role as a parent” and “My social environment sets very high expectations for me as a parent to live up to”) and not to a perception of general pressure on all parents or on the family system. In line with this hypothesis, other studies suggest that an individual’s parenting is influenced by the partner’s feelings about their relationship, rather than his personal concerns (Ponnet et al., 2013; Rousseau & Scharf, 2015). For example, Ponnet et al. (2013) pointed out that marital quality and marital support had partner effects on parenting, while the respective feeling of stress had only actor effects. Future research is needed to shed further light on these questions.

## Limitations and Future Research

There are several limitations that could be addressed in future studies. Due to cross-sectional nature of data, we could only test within-time associations. Longitudinal or experimental research is needed to gain insight into the direction of effects. Further, the sample had characteristics that do not necessarily allow us to generalize our results. The large majority of participating parents were from intact families, that may not be representative of all families with adolescents living in Switzerland (OFS, 2017). Thus, it might be relevant to explore associations between perceived pressure and these three types of involvement in other family constellations, such as single-parent, same-sex families or families struggling with the disability of a parent or child, because these parents may experience societal pressures on parenting in very distinct ways (see e.g., Elliott et al., 2015). Additionally, according to Repetti & Wang (2014) and Whiteman et al. (2003), both parents’ work status (full-time working vs. part-time working vs stay-at-home parent) and family size (one child vs. two or more children) would play a role for understanding these dynamics. In our study, groups were too small to examine their role in a reliable way, but future research should examine the potentially moderating role of these variables. Another limitation of this study was that all variables were only measured through parent’s self-reports (single-informant bias), which may produce stronger associations among the study’s variables. Information from other informants (i.e., multi-informant design) could provide a more complete picture (Van Petegem et al., 2020). The single-informant bias also concerns actor and partner effects, as demonstrated by Orth (2013). Indeed, partner effects may be underestimated because the partner effect is based on measures that have less variance in common than measures on which the actor effect is based (Orth, 2013). As such, further research should use a multiple informants design in order to overcome these limitations and improve the validity of the estimated effects. Finally, in terms of practical implications, future research should also focus on resilience factors that may provide better insights for prevention and intervention efforts. For example, parental mindfulness (e.g., open and receptive attention to and awareness of present moment events and experiences ; Brown & Ryan, 2003), parental self-determination (e.g., one’s tendency to regulate behavior in accordance with one’s personal values, preferences, and interests ; Deci & Ryan, 2012), or parental reflective functioning (e.g., one’s capacity “to hold others’ mind in mind” ; Fonagy et al., 2007, 2018) could help parents to be less susceptible to societal expectations and pressures regarding intensive parenting.

## Conclusion

Despite its limitations, this study contributes to the literature by examining the gendered dimension and the potential negative impact of perceived societal pressure for parents of adolescents. Our results highlighted the greater susceptibility of mothers to feel pressured to be perfect. This suggests that intensive parenting is more actual than ever and can make the parental experience more demanding than it already may be. It is therefore crucial to put into question overly demanding standards that may put parents under pressure. As our results suggest, these pressures may push both parents to become overly involved into their adolescent’s life, hence putting both their own and their child’s mental health at risk. With the aim of promoting adaptive parenting, this study calls for further research on the individual’s factors that may enable parents to better cope with such societal pressure, while at the same time indicates that a stronger policy is needed to encourage more gender equality in the family realm as well.
